# Rainfall Timing as a Key Driver of Cicada Peak Emergence in Urban Habitats

**DOI:** 10.3390/insects17020226

**Published:** 2026-02-22

**Authors:** Jae-Yeon Kang, Yong-Su Kwon, Heejo Lee, Yikweon Jang

**Affiliations:** 1 Ecological Technology Research Team, National Institute of Ecology, 1210 Geumgang-ro, Maseo-myeon, Seocheon-gun 33657, Chungcheongnam-do, Republic of Korea; jaeyoni@nie.re.kr; 2 Department of Life Sciences, Division of Ecoscience, Ewha Womans University, Ewhayeodaegil-52, Seodaemun-gu, Seoul 03760, Republic of Korea; 3 Ecological Bigdata Team, National Institute of Ecology, 1210 Geumgang-ro, Maseo-myeon, Seocheon-gun 33657, Chungcheongnam-do, Republic of Korea; kwonys@nie.re.kr; 4 Protected Area Research Team, National Institute of Ecology, 1210 Geumgang-ro, Maseo-myeon, Seocheon-gun 33657, Chungcheongnam-do, Republic of Korea; forevermed85@nie.re.kr

**Keywords:** cicada, peak emergence, precipitation, synchronous emergence, urban park, urban habitat

## Abstract

Synchronous emergence is a widespread adaptive strategy in cicadas, yet the proximate cues governing its timing in urban environments remain poorly understood. We examined the emergence phenology of three common urban cicada species (*Cryptotympana atrata*, *Hyalessa maculaticollis*, *Graptopsaltria nigrofuscata*) across two urban parks with contrasting habitat structure (a closed-canopy urban forest park vs. an open urban park) in Seoul, South Korea, over three summers (2015–2017). Despite interannual variation in rainfall amount and timing, peak emergence consistently occurred about two weeks after the monsoon rainfall peak. Poisson generalized estimating equation (GEE) analyses confirmed that antecedent precipitation at a 2–3-week lag significantly increased emergence counts across all three species, while precipitation one week prior had no significant effect. Emergence synchrony varied among species and habitat conditions, but the rainfall–emergence lag relationship was robust across years and sites. These findings demonstrate that precipitation timing is a key driver of peak cicada emergence in urban habitats. As East Asia experiences increasingly variable monsoon rainfall under climate change, understanding precipitation-based phenological cues will be essential for predicting the dynamics of urban insect populations.

## Simple Summary

Cicadas time their mass summer emergence with remarkable precision, but the environmental trigger for peak timing remains unclear. By monitoring three cicada species across two urban parks with contrasting habitat types in Seoul, South Korea, we found that emergence peaks consistently occurred about two weeks after monsoon rainfall. This pattern was consistent across species, although the degree of emergence synchrony varied between park types. Our results indicate that precipitation timing is the primary cue for peak emergence. As monsoon rainfall becomes increasingly unpredictable under climate change, these findings provide important insight into how urban cicada populations may respond to future shifts in precipitation regimes.

## 1. Introduction

Synchronous emergence is the coordinated mass appearance of adult insects within a short time window and is a common life-history strategy across diverse insect taxa. It is shaped by evolutionary pressures such as predator satiation [1,2], which reduces individual predation risk, and reproductive synchrony [3,4], which maximizes mating opportunities with a narrow timeframe. In some cases, like periodical cicadas, additional strategies such as predator cycle avoidance further enhance fitness [5,6]. These synchronized events depend on environmental cues to signal appropriate timing, ensuring that cohorts emerge under favorable conditions.

Precipitation has been increasingly recognized as a proximate cue shaping the timing and synchrony of insect emergence events. Rainfall can facilitate emergence by softening soil and enabling burrowing of final-instar nymphs [7], and by providing humid conditions essential for successful ecdysis [8]. However, excessive rainfall can be detrimental, increasing nymphal mortality or damaging newly emerged adults [9]. Rainfall events typically precede emergence peaks by days to weeks [10], and in arid environments, emergence may be synchronized by cumulative precipitation thresholds across multiple years [11]. Whether precipitation timing specifically governs peak emergence—the period of maximum population-level synchrony—has received less attention, particularly in urban environments where habitat structure may differentially influence how precipitation affects emergence conditions.

Cicadas are particularly well suited for examining precipitation as a proximate trigger of emergence phenology. Their multi-year subterranean development integrates long-term thermal signals—with accumulated heat during late spring governing developmental readiness [12,13]—while final emergence timing may respond to short-term environmental cues such as soil moisture availability [8]. In periodical cicadas, emergence is triggered when soil temperature exceeds specific thresholds (~18 °C) [14], but the precise timing of mass emergence events may depend on additional cues such as precipitation. This suggests that once thermal requirements are met, precipitation may play a key role in determining when peak emergence occurs—a hypothesis that remains largely untested in urban environments. Here, we focus on peak emergence timing rather than first emergence, as peak timing better reflects the coordinated reproductive period critical for predator satiation and mating success [15,16].

Urban environments, where fragmented green spaces create diverse habitat structures, offer a natural setting to examine how precipitation shapes emergence phenology across different ecological conditions. Urbanization can create habitat heterogeneity across park types [17] through increased impervious surfaces and modified drainage systems. In Seoul, South Korea, urban parks range from densely forested sites with multi-layered vegetation to open, lawn-dominated parks with minimal tree cover. These contrasting structures create distinct habitat conditions, including vegetation density and ground cover, that may differentially influence how regional rainfall affects local emergence environments. Previous urban cicada studies in Seoul documented species composition and abundance patterns [18,19,20], but the phenology of emergence—particularly its timing and synchrony—has received limited attention. Whether precipitation timing governs peak emergence across contrasting urban habitats, and whether co-occurring species respond differentially, remains unclear.

We investigated peak emergence of three cicada species (*Cryptotympana atrata*, *Hyalessa maculaticollis*, and *Graptopsaltria nigrofuscata*) across two urban parks with contrasting habitat types (a closed-canopy urban forest park vs. an open urban park) in Seoul over three consecutive summers (2015–2017). Using weekly emergence surveys and precipitation data from nearby weather stations, we addressed two questions: (1) Does precipitation timing predict peak emergence timing consistently across years and habitat types? (2) Do the three cicada species respond uniformly or differentially to precipitation, and does emergence synchrony vary with habitat type?

## 2. Materials and Methods

### 2.1. Study Sites

**Figure 1:**
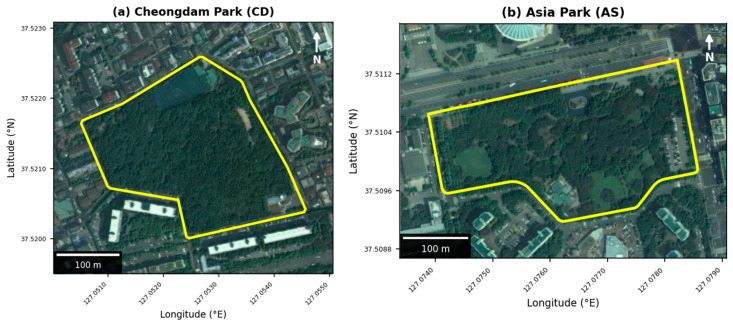
Study site locations with aerial imagery: (**a**) Cheongdam Park (CD) and (**b**) Asian Park (AS) in Seoul, South Korea. Yellow lines denote park boundaries (source: Seoul Open Data Portal). Base imagery: National Geographic Information Institute.

This study was conducted in Seoul, the capital of South Korea, a densely urbanized metropolis with about 9.6 million residents, characterized by numerous buildings and roads. To investigate cicada emergence patterns under differing ecological conditions within the same urban climate regime, we selected two urban parks with contrasting vegetation structure: Cheongdam Park (CD) and Asian Park (AS). These sites are located approximately 2km apart (straight-line distance), ensuring similar regional meteorological conditions. However, they differ substantially in vegetation structure and land use, providing a natural comparison between contrasting habitat types (Figure 1).

CD (37°31′12.02″ N, 127°03′12.01″ E) is a 5.8 ha urban forest park situated on a low hill. It retains more natural topography and supports dense, continuous canopy cover. Dominant tree species include *Robinia pseudoacacia* (29.7% of total area), *Quercus mongolica* (23.5%), and *Populus tomentiglandulosa* (19.5%) [21]. The site is managed for conservation and ecological integrity, with minimal disturbance and little human traffic.

AS (37°30′38.04″ N, 127°04′29.00″ E) is a typical 6.6 ha urban park characterized by flat terrain and open spaces. Approximately 69% of the park is managed green space, with scattered plantings of *Pinus densiflora*, *Prunus serrulata*, *Zelkova serrata*, and *Platanus orientalis*. Due to its sparse canopy and proximity to sports facilities, AS experiences high solar exposure and relatively warm conditions.

### 2.2. Study Species and Field Sampling

Approximately 12 cicada species are recorded in South Korea, of which 6 are commonly encountered in Seoul Metropolitan City. In this study, we initially monitored six species across two urban sites: *Cryptotympana atrata*, *Hyalessa maculaticollis, Meimuna mongolica*, *Meimuna opalifera*, *Graptopsaltria nigrofuscata*, and *Platypleura kaempferi* [18,19]. However, due to extremely low densities (Table S1), *M. mongolica*, *M. opalifera*, and *P. kaempferi* (mean occurrence < 0.08 individuals per quadrat across all site-year-week combinations (*n* = 54)) were excluded from subsequent statistical analyses. Thus, the final analysis focused on three abundant and consistently detected species: *C. atrata*, *H. maculaticollis*, and *G. nigrofuscata*. These species typically emerge between late June and August, aligning with the study’s 9-week sampling window, which spanned from the last week of June (week 1) to the last week of August (week 9) each year from 2015 to 2017. Weekly sampling over this 9-week period was considered adequate for capturing population-level peak emergence patterns, as weekly plot sampling has been shown to effectively characterize emergence phenology and detect seven-day differences in peak timing between habitats [22].

To quantify cicada emergence, we used exuviae (shed exoskeletons) as a proxy for newly emerged individuals. Unlike adults, exuviae remain attached to substrates and are unaffected by predation, weather, or subsequent dispersal, making them reliable indicators of emergence timing and density [23]. Exuviae were collected shortly after emergence to ensure accurate species identification and minimize loss due to environmental exposure.

A quadrat-based sampling method was employed to assess emergence density, following Kang et al. [24,25]. Circular quadrats (20 m in diameter) were centered on focal host trees to minimize edge effects [26,27]. Five circular quadrats were established at each study site (AS: *n* = 5, CD: *n* = 5), with placement designed to capture within-site habitat variability. Species identification was conducted using morphological characteristics of exuviae, following the identification key developed by Lee et al. [18]. This method ensured taxonomically robust classification of species across all surveys.

To characterize habitat conditions at each sampling point, we measured several environmental variables across the study period. Altitude for each point was extracted from Google Earth Pro (Google LLC, Mountain View, CA, USA) based on GPS coordinates. Tree density was counted once in 2015, and diameter at breast height (DBH; 1.3 m above ground) was measured with three replicates per tree using a measuring tape. Canopy cover and percent cover of arbor, shrub, and grass layers were measured at least three times in 2016 within quadrats centered on each point, and mean values were used for analyses. Canopy cover estimation followed the ocular estimation method in the USDA Forest Service Forest Inventory and Analysis (FIA) field manual (version 3.0) with standard reference cards [28]. Soil hardness was measured in 2017 using a hand-push penetrometer (Yamanaka type, No. 351, Fujiwara Scientific, Tokyo, Japan) following Moriyama & Numata [7]. Ten repeated measurements were taken within each quadrat on non-rainy days to avoid precipitation effects on soil moisture.

Precipitation data (mm) were obtained from the Korea Meteorological Administration (http://data.kma.go.kr, accessed on 2 October 2017) at the Songpa-gu AWS for AS and the Gangnam-gu AWS for CD. To characterize temporal variation, weekly means of precipitation (TOT_Precip; mm) were calculated by averaging daily values within each calendar week, covering a 9-week observation window from late June (Week 1) to late August (Week 9) for each study year (2015–2017). These variables were subsequently used to evaluate interannual and inter-site differences and to model their effects on cicada emergence patterns.

### 2.3. Statistical Analyses

We analyzed weekly data collected over nine weeks (late June through late August) from 2015 to 2017 at both parks (AS and CD) to assess the effects of meteorological conditions on cicada emergence. Prior to parametric testing, normality was assessed using the Shapiro–Wilk tests. Precipitation violated normality assumptions (*p* < 0.001) and was log-transformed [log(x + 1)] prior to parametric analysis. Habitat characteristics were compared between sites using independent samples *t*-tests. Annual and spatial variations in precipitation was analyzed using two-way ANOVA to evaluate main effects of year, site, and their interaction, followed by Tukey’s HSD post hoc tests when significant. To quantify temporal characteristics of peak emergence patterns, we used the interquartile range (IQR) as an index of emergence synchrony. IQR was calculated as the range of weeks corresponding to the 25th to 75th percentiles of cumulative emergence for each species–year–site combination, with smaller values indicating higher synchrony.

To quantitatively assess the relationship between antecedent precipitation and cicada emergence abundance, Poisson generalized estimating equations (GEEs) were used to model weekly emergence counts as a function of prior-week precipitation. Emergence counts for each species were calculated as the sum of individuals across five quadrats per site per week. GEEs were employed to account for the repeated-measures structure of weekly observations nested within site–year clusters (*n* = 6 clusters: 2 sites × 3 years), using an exchangeable working correlation structure and a Poisson distribution with log link function. Independent variables were total weekly precipitation recorded 1 week (lag1), 2 weeks (lag2), and 3 weeks (lag3) before each observation week. For example, for an observation at week *t*, lag2 represents precipitation at week *t* − 2. Lag effects were calculated only within the same year. As the study period began at week 1, lag1 was available from week 2, lag2 from week 3, and lag3 from week 4. Separate models were fitted for each species and lag interval to evaluate lag-specific effects. Data were pooled across years and sites to ensure adequate sample size, with the GEE clustering structure accounting for within-cluster correlations. Model results are reported as regression coefficients (β), incidence rate ratios (IRR = e^β^) with 95% confidence intervals, and Wald test *p*-values. IRR represents the multiplicative change in expected emergence counts per 1 mm increase in antecedent precipitation.

All statistical analyses and visualizations were performed using Python v3.12.4 (Python Software Foundation) in the Jupyter Notebook environment (v7.0.8, Anaconda distribution). We used the statsmodels library (v0.14.5) for Poisson GEE modeling and matplotlib (v3.10.6) and seaborn (v0.13.2) libraries for visualization.

## 3. Results

### 3.1. Habitat Characteristics

Although both parks were located close to each other, they exhibited substantial differences in habitat structure (Table 1). The forested park (CD) was situated at significantly higher altitude than the urban park (AS), with nearly three times higher tree density, and showed significantly higher arbor cover and shrub cover, and tended to have higher canopy cover. In contrast, AS featured trees with significantly larger DBH and higher grass cover, though the latter was not statistically significant. Soil hardness did not differ significantly between sites. These contrasting structures indicate that AS represents an open habitat dominated by scattered large trees, whereas CD exhibits a closed, multi-layered forest structure.

### 3.2. Interannual Variation in Precipitation and Cicada Emergence

Two-way ANOVA on precipitation revealed no significant year (*F*(2,48) = 2.68, *p* = 0.079) or site effects (*F*(1,48) = 0.00, *p* = 0.97). Nevertheless, mean weekly precipitation in 2017 (AS: 94.5 mm, CD: 81.5 mm) was descriptively higher than in 2015 (AS: 31.1 mm, CD: 30.6 mm) and 2016 (AS: 36.2 mm, CD: 42.1 mm), with greater weekly variability (SD > 100 mm in 2017 vs. SD < 50 mm in earlier years).

Across the two sites, a total of 4845 exuviae of the three cicada species were collected over the three-year study period (Table 2). Total emergence abundance increased from 1275 individuals in 2015 to 2049 in 2017, but this trend was not statistically significant (one-way ANOVA: *F*(2, 51) = 0.51, *p* = 0.60). Species-specific habitat preferences were significant (*t*-test, all *p* < 0.01): *C. atrata* was more abundant at AS, whereas *H. maculaticollis* and *G. nigrofuscata* showed higher abundance at CD (Table 2).

### 3.3. Annual and Spatial Variation in Peak Emergence Patterns

**Figure 2:**
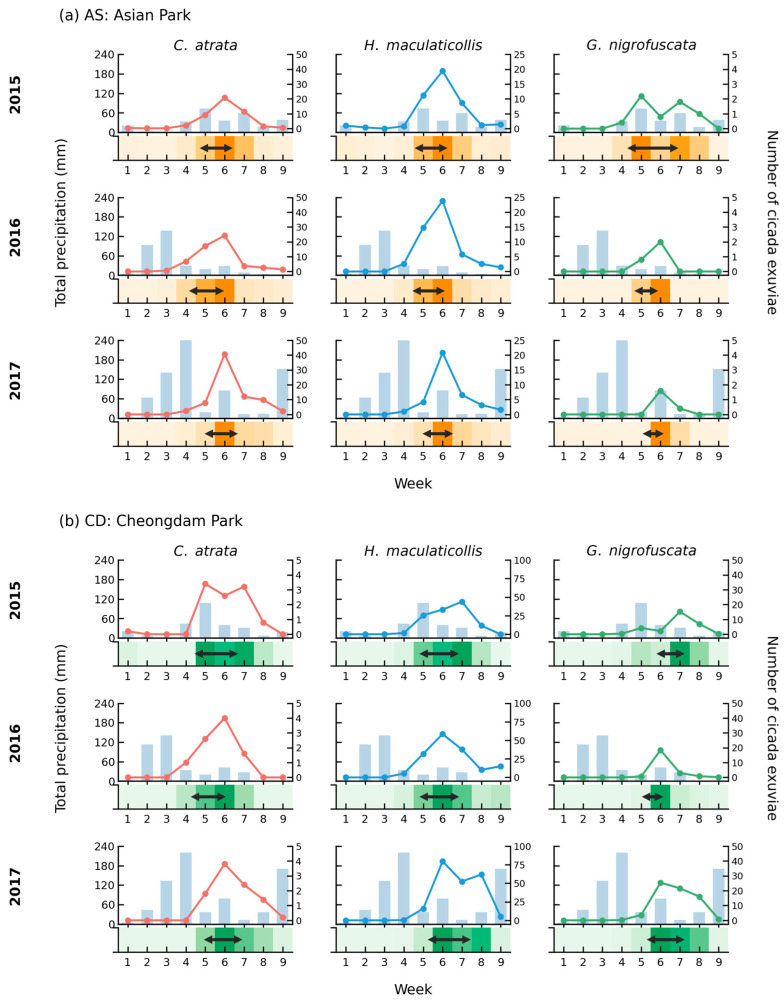
Comparison of weekly cicada emergence peaks and associated precipitation patterns for three species—*C. atrata*, *H. maculaticollis*, and *G. nigrofuscata*—in two urban parks: (**a**) AS (Asian Park) and (**b**) CD (Cheongdam Park). Panels are arranged in a 3 × 3 grid (rows: 2015–2017; columns: species). Within each panel, bars show weekly total precipitation (mm; left y-axis) and lines show the number of cicada exuviae collected (right y-axis). Line colors correspond to each species (red: *C. atrata*; blue: *H. maculaticollis*; green: *G. nigrofuscata*). The bottom heat strip represents weekly emergence intensity normalized to the 0–1 range using min–max scaling, with orange indicating AS and green indicating CD. Arrow bars indicate the interquartile range (IQR) of emergence weeks. Weekly units cover nine study weeks (late June–late August) for each year.

Cicada emergence patterns varied markedly across years, corresponding with differences in precipitation timing, amount (Figure 2). In 2015, precipitation peaked late in week 5 with relatively low amounts (AS: 73.0 mm; CD: 107.5 mm), characteristic of a delayed and weak monsoon. Emergence was relatively dispersed, with IQRs of 1.0–2.2 weeks across species—the widest among the three years (Table S2).

In 2016, precipitation peaked two weeks earlier in week 3 with substantial amounts (AS: 136.9 mm; CD: 140.4 mm). Correspondingly, emergence timing advanced across all species and sites: overall median emergence was earliest among the three years (5.39 weeks). This advancement was more pronounced at CD (6.00 → 5.52 weeks, 3.4 days earlier) than at AS (5.53 → 5.26 weeks, 1.9 days earlier). In 2017, total precipitation was highest (AS: 850.2 mm; CD: 733.6 mm), with a peak in week 4 followed by sustained rainfall throughout the season. Median emergence was 5.86 weeks, later than in 2016, and IQRs ranged from 0.6 to 1.7 weeks.

Across all three years, the temporal relationship between precipitation and emergence was evident. Precipitation peaks preceded maximum emergence by 2–3 weeks at both sites. Early peak precipitation in 2016 (week 3) was associated with the earliest emergence (median 5.39 weeks) and highest synchrony (IQR 0.6–1.6 weeks). In contrast, late peak precipitation in 2015 (week 5) was associated with the lowest synchrony (IQR 1.0–2.2 weeks). In 2017, total precipitation was the highest with sustained rainfall, and IQRs (0.6–1.7 weeks) were intermediate between 2015 and 2016.

The temporal relationship between precipitation and emergence was consistent across both sites, but emergence synchrony differed between parks. For *C. atrata*, both sites exhibited similar IQR patterns, ranging from 1.2 to 1.8 weeks across years, indicating comparable synchrony regardless of habitat type. In contrast, *H. maculaticollis* showed consistently broader IQRs at CD (1.55–1.74 weeks) than at AS (1.11–1.24 weeks) across all three years, indicating lower emergence synchrony in the forested park. In particular, at CD in 2017, emergence extended from weeks 6 through 8 (79.4, 52.4, and 61.8 individuals, respectively), showing the widest IQR (1.74 weeks) among all year–site combinations.

### 3.4. Predictive Influence of Antecedent Precipitation on Cicada Emergence

**Figure 3:**
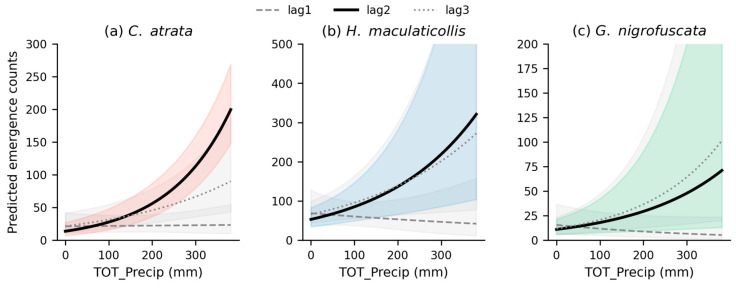
Predicted emergence counts as a function of antecedent precipitation based on Poisson generalized estimating equations (GEEs) for three cicada species: (**a**) *C. atrata*, (**b**) *H. maculaticollis*, and (**c**) *G. nigrofuscata*. Curves represent model-predicted counts for each lag interval (dashed gray: lag1, 1 week prior; solid black: lag2, 2 weeks prior; dotted gray: lag3, 3 weeks prior). Shaded bands indicate 95% confidence intervals, with species-colored bands for lag2 and gray bands for lag1 and lag3.

Poisson GEE analysis revealed that antecedent precipitation significantly predicted cicada emergence counts, with the strongest and most consistent effects at a 2-week lag (Table 3, Figure 3). Precipitation two weeks prior (lag2) significantly increased emergence counts for all three species: *C. atrata* (β = 0.0070, IRR = 1.007, 95% CI: 1.006–1.008, *p* < 0.001), *H. maculaticollis* (β = 0.0047, IRR = 1.005, 95% CI: 1.002–1.007, *p* < 0.001), and *G. nigrofuscata* (β = 0.0049, IRR = 1.005, 95% CI: 1.001–1.008, *p* = 0.006). These IRR values indicate that each additional 100 mm of precipitation two weeks prior was associated with approximately 2-fold higher emergence in *C. atrata* (+102%), and 60–63% higher emergence in *H. maculaticollis* and *G. nigrofuscata*.

Precipitation three weeks prior (lag3) was also significant for all three species, though with smaller effect sizes: *C. atrata* (β = 0.0038, *p* = 0.001), *H. maculaticollis* (β = 0.0037, *p* = 0.014), and *G. nigrofuscata* (β = 0.0057, *p* = 0.002). Precipitation one week prior (lag1) showed no significant effect for any species (all *p* > 0.10). The consistent significance of lag2 across all three species, combined with the absence of lag1 effects, supports a precipitation–emergence lag of approximately two weeks.

## 4. Discussion

The most striking finding of this study is that peak emergence of urban cicadas consistently occurred with a 2–3-week lag following precipitation peaks. This suggests that precipitation acts as a proximate trigger for the timing of peak emergence in final-instar nymphs. In this study, we focused on peak emergence timing rather than emergence initiation, as peak timing reflects the population-level response to environmental cues and is more relevant for predicting the period of maximum adult activity.

A similar pattern of emergence approximately 30 days after precipitation has been reported in urban cicadas in Japan [10]. The lag effect of cicada peak emergence following precipitation can be interpreted as precipitation improving emergence conditions for nymphs. Precipitation can physically facilitate surface excavation and movement of final-instar nymphs by softening soil structure and reducing mechanical resistance [7], while high atmospheric humidity provides essential conditions for post-emergence molting and exoskeleton hardening. Since cicada nymphs are extremely vulnerable to desiccation during emergence [29], the moist conditions provided by antecedent precipitation serve as a key factor ensuring successful emergence.

Beyond the timing of emergence, the degree of emergence synchrony also varied among years. Emergence synchrony was relatively high when monsoon rainfall was both abundant and peaked early (2016, 2017), and lowest when rainfall was scarce and delayed (2015). Total emergence abundance also tended to be higher in wetter years, though not statistically significant. Positive relationships between precipitation and cicada emergence have been documented in both tropical forests, where cicada exuviae abundance correlates with rainfall [30], and arid regions, where multi-year precipitation thresholds govern emergence timing [13]. These patterns suggest that precipitation may influence not only the timing and synchrony of emergence but also overall emergence success. However, generalization is limited with only three years of data, and long-term studies are needed given that climate change is predicted to increase variability in precipitation patterns.

Comparison across the two sites and three species revealed both general patterns and species-specific variation in the precipitation–emergence relationship. The two sites differed in habitat type, with AS representing an open park with patchy tree cover and CD a closed-canopy forest. *C. atrata* showed consistent emergence concentration and comparable lagged precipitation effects at both sites, suggesting that the precipitation–emergence lag pattern for this species was consistent across the two sites in our study. *H. maculaticollis* also exhibited significant lagged precipitation effects, but its emergence period was consistently broader at CD. This may reflect differences in post-rain moisture retention between sites: the closed-canopy forest at CD may sustain near-surface moisture longer than the more open AS, where exposed areas tend to dry faster after rainfall [31]. *G. nigrofuscata* showed significant precipitation responses, although its low abundance at AS limited robust site-level comparison. Together, these results suggest that although the direction and lag structure of precipitation effects were broadly similar among the species examined, the temporal spread of emergence differed with habitat type, potentially reflecting site-level differences in post-rain microhabitat conditions.

There are several limitations in this study. The survey period was limited to three years with only two study sites, and weekly surveys could not capture fine-scale daily emergence patterns. In addition, we did not directly measure soil moisture dynamics, which limits mechanistic inference about the hydrological pathway linking precipitation to emergence. Nonetheless, the consistent reproduction of the approximately 2–3-week lag pattern after precipitation peaks across all three years, both sites, and all three species represents a key strength of this study. This reproducibility demonstrates the robustness of the precipitation–emergence relationship, and future validation across longer periods and diverse habitats is needed to establish the generality of this relationship. Daily or higher temporal resolution observations would also enable more precise characterization of the physiological response time from precipitation to emergence.

## 5. Conclusions

This study demonstrates that antecedent precipitation acts as the primary proximate cue for peak emergence of urban cicadas, with a consistent 2–3-week lag across sites and species. Although this general precipitation–emergence relationship was robust, modest interspecific variation in emergence synchrony was observed across contrasting urban habitat types. Importantly, our findings indicate that the temporal concentration of rainfall governs emergence synchrony in fragmented urban landscapes. Given increasing variability and localization of monsoon rainfall under climate change in East Asia, shifts in precipitation timing may substantially alter urban insect phenology. Beyond cicadas, these results provide a framework for incorporating precipitation-based cues into climate-driven emergence prediction models for urban insects.

## Figures and Tables

**Table 1:** Habitat characteristics of two study sites in Seoul, South Korea. Values are mean ± standard deviation (*n* = 5 quadrats per site). Independent *t*-tests were performed to compare habitat variables between Asian Park (AS) and Cheongdam Park (CD). * *p* < 0.05, ** *p* < 0.01, *** *p* < 0.001.

Variable	AS	CD	*t*	*p*
Altitude (m)	17.9 ± 2.6	62.8 ± 11.2	−8.756	<0.001 ***
Tree density(trees/m^2^)	0.028 ± 0.007	0.076 ± 0.014	−6.839	<0.001 ***
DBH (cm)	110.0 ± 15.0	68.6 ± 11.6	4.890	0.001 **
Canopy cover (%)	71.0 ± 11.4	83.0 ± 7.6	−1.960	0.086
Arbor cover (%)	18.0 ± 6.7	34.0 ± 4.2	−4.525	0.002 **
Shrub cover (%)	10.0 ± 7.9	28.0 ± 12.5	−2.714	0.027 *
Grass cover (%)	19.0 ± 23.8	9.0 ± 4.2	0.925	0.382
Soil hardness (kg/cm^2^)	3.2 ± 1.0	2.6 ± 1.5	0.766	0.466

**Table 2:** Total emergence counts by species, sites, and year (2015–2017).

Site	Species	2015	2016	2017
AS	*C. atrata*	234	283	374
	*H. maculaticollis*	220	255	187
	*G. nigrofuscata*	31	14	10
	Subtotal	495	562	584
CD	*C. atrata*	51	46	48
	*H. maculaticollis*	579	791	1073
	*G. nigrofuscata*	146	115	338
	Subtotal	780	959	1465
	Total	1275	1521	2049

**Table 3:** Poisson generalized estimating equation (GEE) results for the effect of antecedent precipitation on weekly cicada emergence counts. Emergence counts represent the sum of individuals across five quadrats per site per week. An exchangeable working correlation structure was used with site–year clusters (*n* = 6). IRR = incidence rate ratio (e^β^); CI = confidence interval. * *p* < 0.05, ** *p* < 0.01, *** *p* < 0.001.

Species	Lag	*n*	β	SE	IRR	95% CI	*p*-Value
*C. atrata*	lag1	48	0.0002	0.0014	1.000	0.997–1.003	0.862
	lag2	42	0.0070	0.0007	1.007	1.006–1.008	<0.001 ***
	lag3	36	0.0038	0.0012	1.004	1.001–1.006	0.001 **
*H. maculaticollis*	lag1	48	−0.0013	0.0022	0.999	0.994–1.003	0.548
	lag2	42	0.0047	0.0013	1.005	1.002–1.007	<0.001 ***
	lag3	36	0.0037	0.0015	1.004	1.001–1.007	0.014 *
*G. nigrofuscata*	lag1	48	−0.0028	0.0017	0.997	0.994–1.001	0.103
	lag2	42	0.0049	0.0018	1.005	1.001–1.008	0.006 **
	lag3	36	0.0057	0.0019	1.006	1.002–1.009	0.002 **

## Data Availability

The datasets generated and analyzed during the current study are available in the Supplementary Materials. Other data are not publicly available due to institutional restrictions but are available from the authors upon reasonable request.

## Supplementary Materials

The following supporting information can be downloaded at https://www.mdpi.com/article/10.3390/insects17020226/s1. Table S1: Quadrat-level cicada emergence counts by species, site, and week (2015–2017); Table S2: Emergence timing IQR values by species, site, and year.

## Abbreviations

IQR	Interquartile Range
GEE	Generalized Estimating Equation
IRR	Incidence Rate Ratio
